# ANISEED 2019: 4D exploration of genetic data for an extended range of tunicates

**DOI:** 10.1093/nar/gkz955

**Published:** 2019-11-04

**Authors:** Justine Dardaillon, Delphine Dauga, Paul Simion, Emmanuel Faure, Takeshi A Onuma, Melissa B DeBiasse, Alexandra Louis, Kazuhiro R Nitta, Magali Naville, Lydia Besnardeau, Wendy Reeves, Kai Wang, Marie Fagotto, Marion Guéroult-Bellone, Shigeki Fujiwara, Rémi Dumollard, Michael Veeman, Jean-Nicolas Volff, Hugues Roest Crollius, Emmanuel Douzery, Joseph F Ryan, Bradley Davidson, Hiroki Nishida, Christelle Dantec, Patrick Lemaire

**Affiliations:** 1 CRBM, Université de Montpellier, CNRS, Montpellier, France; 2 Bioself Communication; 28 rue de la Bibliothèque, F-13001 Marseille, France; 3 ISEM, Université de Montpellier, CNRS, IRD, EPHE, Montpellier, France; 4 Laboratoire d’Informatique de Robotique et de Microélectronique de Montpellier (LIRMM), Université de Montpellier, CNRS, Montpellier, France; 5 Department of Biological Sciences, Graduate School of Science, Osaka University, 1-1 Machikaneyama-cho, Toyonaka, Osaka 560-0043, Japan; 6 Whitney Laboratory for Marine Bioscience, 9505 Ocean Shore Boulevard, St. Augustine, FL 32080, USA; 7 Department of Biology, University of Florida, 220 Bartram Hall, Gainesville, FL 32611, USA; 8 DYOGEN, IBENS, Département de Biologie, Ecole Normale Supérieure, CNRS, Inserm, PSL Research University, F-75005 Paris, France; 9 IBDM, Université Aix-Marseille, CNRS, Marseille, France; 10 Institut de Génomique Fonctionnelle de Lyon, Université de Lyon, Ecole Normale Supérieure de Lyon, Université Claude Bernard Lyon 1, CNRS; 46 allée d’Italie, F-69364 Lyon, France; 11 Laboratoire de Biologie du Développement de Villefranche-sur-mer (LBDV), Sorbonne Universités, Université Pierre-et-Marie-Curie, CNRS; Quai de la Darse, F-06234 Villefranche-sur-Mer Cedex, France; 12 Division of Biology, Kansas State University, Manhattan, KS 66506, USA; 13 State Key Laboratory of Cell Biology, Shanghai Key Laboratory of Molecular Andrology, CAS Center for Excellence in Molecular Cell Science, Institute of Biochemistry and Cell Biology, Chinese Academy of Sciences, University of Chinese Academy of Sciences, 320 Yueyang Road, Shanghai 200031, China; 14 Université de Montpellier, Montpellier, France; 15 Department of Chemistry and Biotechnology, Faculty of Science and Technology, Kochi University, Kochi-shi, Kochi, Japan; 16 Department of Biology, Swarthmore College, Swarthmore, PA 19081, USA

## Abstract

ANISEED (https://www.aniseed.cnrs.fr) is the main model organism database for the worldwide community of scientists working on tunicates, the vertebrate sister-group. Information provided for each species includes functionally-annotated gene and transcript models with orthology relationships within tunicates, and with echinoderms, cephalochordates and vertebrates. Beyond genes the system describes other genetic elements, including repeated elements and *cis*-regulatory modules. Gene expression profiles for several thousand genes are formalized in both wild-type and experimentally-manipulated conditions, using formal anatomical ontologies. These data can be explored through three complementary types of browsers, each offering a different view-point. A developmental browser summarizes the information in a gene- or territory-centric manner. Advanced genomic browsers integrate the genetic features surrounding genes or gene sets within a species. A Genomicus synteny browser explores the conservation of local gene order across deuterostome. This new release covers an extended taxonomic range of 14 species, including for the first time a non-ascidian species, the appendicularian *Oikopleura dioica*. Functional annotations, provided for each species, were enhanced through a combination of manual curation of gene models and the development of an improved orthology detection pipeline. Finally, gene expression profiles and anatomical territories can be explored in 4D online through the newly developed Morphonet morphogenetic browser.

## INTRODUCTION

Tunicates are marine invertebrates with a key phylogenetic position as the sister group of the vertebrates (1,2). Three major groups of tunicates have been classically described. The sessile ascidians form the largest group with several thousand species listed. Two additional groups of tunicates have a pelagic life-style and rapid molecular evolution rates, the thaliaceans and the appendicularians. Their phylogenetic position with respect to ascidians has long remained debated. Molecular phylogenies suggest that the fast-evolving appendicularians are the sister group of all other tunicates, and that thaliaceans form a monophyletic group nested within ascidians (3,4)

Tunicates studies have led to important discoveries in a variety of scientific fields. They illuminated the origin of vertebrate features, including the neural crest (5) or the secondary heart field (6,7). The simplicity of ascidian embryos makes them ideal to decipher the regulatory networks controlling embryonic development (8–10) and their evolution within the taxon (11–14). Colonial ascidians have striking regenerative capacities, including Whole Body Regeneration from a small number of vascular cells (15–17). Some tunicates also have an important function in marine ecosystems (18) or can be damaging invasive species (19). They can finally be used to study the response of the marine fauna to global climate change (20) or to monitor pollution (21). Unlocking the potential of tunicate research across many fields requires the development of a suitable computational framework to centralize molecular, taxonomic and ecological information.

ANISEED is the main model organism database for the worldwide community of scientists working on tunicates, the sister-group of vertebrates (22–24). Established 15 years ago, the system has grown to become a fundamental resource for this community of around eighty labs worldwide, mostly located in Europe, Japan and the USA. On average in 2018, 170 000 pages were visited each month by roughly 1700 unique visitors, coming from all main international ascidian labs.

The ANISEED 2017 release (24) covered 10 species and integrated for each species: (i) a taxonomy page with suitable links to external taxonomic, ecological and molecular resources; (ii) a main knowledge base, the ‘Developmental browser’ structured around extended functional, gene expression and anatomical ontologies and interactive gene phylogenies as a comparative framework to study the developmental programs of different species; (iii) a multispecies genomic browser to visualize the position of genetic features along chromosomes; (iv) a Genomicus synteny browser (25) to analyse the evolution of gene order across tunicate and other chordate genomes. Care was taken during the development of ANISEED that the tool remains generic and adaptable with minimal effort to any developmental model organism.

During the preparation of ANISEED 2019, we added three additional solitary or colonial ascidian species with recently sequenced genome: *Molgula occulta*, *Corella inflata* and *Botryllus leachii* and extended for the first time the system to a non-ascidian species, the appendicularian *Oikopleura dioica*. We significantly improved the functional annotation of genes, through the manual curation of gene model sets in some species and the refinement of our orthology assignment procedure, which now detects vertebrate orthologs for a majority of genes from all ascidians species, including the main ascidian model species, *Ciona robusta* (formerly referred to as *Ciona intestinalis* type A). We enriched the genomics datasets related to the control of gene expression in existing and new species. Finally, we interfaced the developmental browser of ANISEED 2019 to the MORPHONET morphogenetic browser, allowing 4D exploration of gene expression profiles.

### Extension of the taxonomic range covered

In addition to the ten ANISEED 2017 species, three new ascidian species, for which genome and gene models were recently made available, were added to the portal. The solitary stolidobranch *Molgula occulta* is so closely related to *M. oculata* that hybrids between these two species can be produced, yet it is one of the few ascidian species that gives rise to tail-less larvae (26). The solitary Corellidae phlebobranch *Corella inflata*, is a distant relative of Cionidae (*Ciona* species) and Ascididiae (*Phallusia* species), which can be efficiently electroporated (B. Davidson, personal communication). The third species is a colonial stolidobranch species, *Botrylloides leachii*, closely related to *Botryllus schlosseri*, but with a much smaller genome size (27). Its regenerative potential is such that it is capable of whole body regeneration (WBR), including the germline, from a tiny piece of vascular tissue (28).

**Figure 1.**
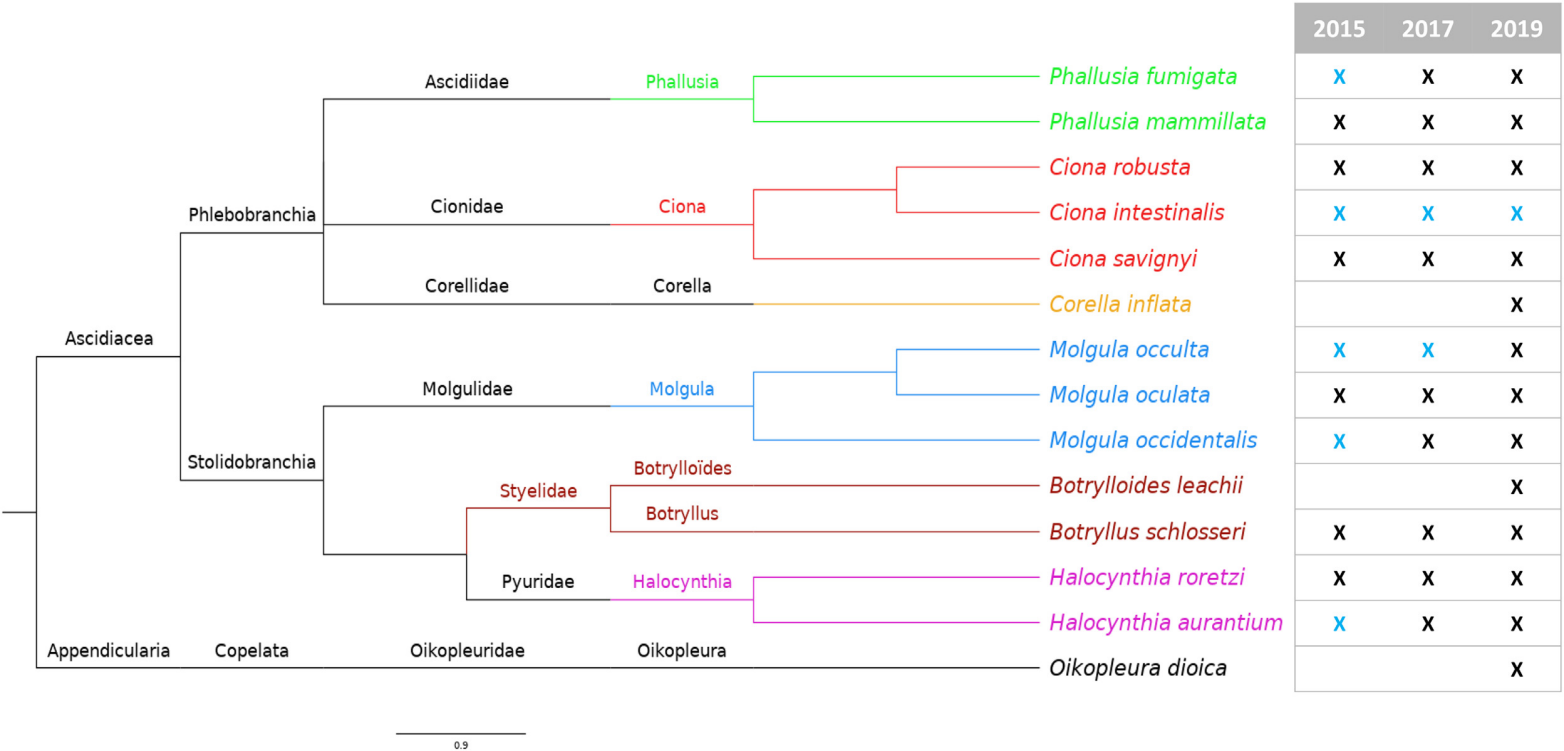
Evolution of the species covered by successive ANISEED releases. A blue cross indicates a species covered by the ANISEED genome browser only, without dedicated section in the developmental browser. A black cross indicates a species fully covered by the system.

ANISEED 2019 now also covers for the first time a second tunicate group: the Appendicularia, which retain a tadpole morphology throughout their short adult life (29). A high-quality genome assembly was recently generated from a Japanese isolate of this species, which was annotated using the ANISEED annotation pipeline and can be explored through a dedicated genome browser and a section of the developmental browser. Aplousobranchs and thaliaceans are currently not represented as no sequenced genome of sufficient quality have been reported for these groups (Figure 1).

### Improved functional annotation pipeline

To improve functional annotations, we first curated the gene model sets retrieved from the various genome projects. The main improvement was achieved for *Ciona robusta* (formerly referred to as *Ciona intestinalis type A*), for which we completed the KH2012 gene model set with 1247 NCBI models for genes that had been missed in the KH set. In *Phallusia mammillata*, 724 inaccurate transcripts for 672 gene models were suppressed and the strand of 81 transcripts was reverted. Besides coding genes, repeats elements were manually reannotated.

**Figure 2.**
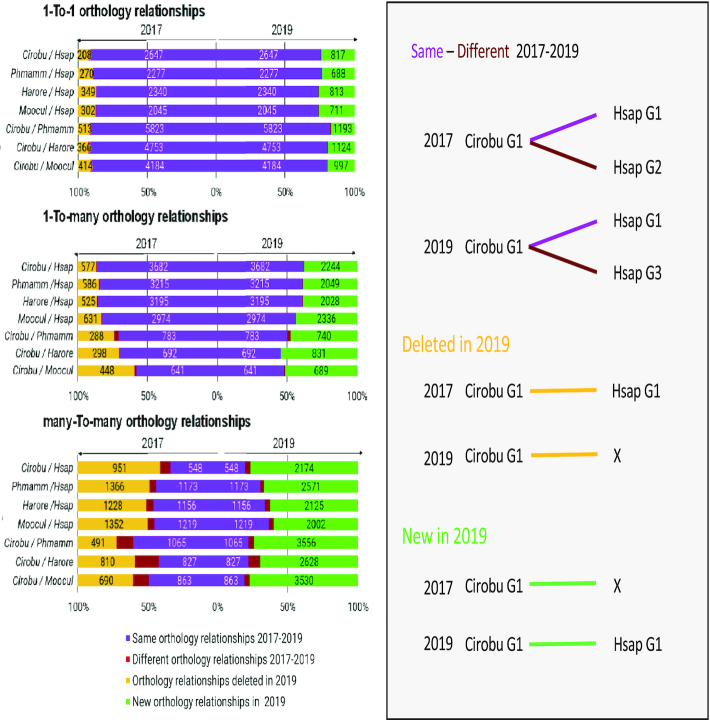
Comparison of the one-to-one, one-to-many and many-to-many orthology relationships detected across the whole genome in the ANISEED 2017 and 2019 releases. Each half of the bar graphs present the analysis in 2017 (left half) and 2019 (right half) of the percentage (and number) of orthology relationships linking one *Ciona robusta (Cirobu*) gene to one gene in the second species indicated (*Phmamm: Phallusia mammillata; Harore: Halocynthia roretzi; Moocul; Molgula oculata; Hasp: Homo sapiens)*. Four scenarios are distinguished, as illustrated on the right side of the figure. Same orthology relationships 2017–2019: orthology relationships found with both the 2017 and 2019 orthology pipelines. Different orthology relationships 2017–2019: the *Cirobu* gene has orthologs in the second species according to both pipelines, but these orthologs differ. New orthology relationships in 2019: 2019 orthology relationships linking a *Cirobu* gene, without 2017 ortholog in the second species, to one or more orthologs in this species. A minority of these cases (e.g. 35 in Cirobu/Hsap one-to-one) correspond to orthology relationships for *Cirobu* NCBI gene models that were added to complement the KH gene model set. Deleted orthology relationships in 2019: 2017 orthology relationships linking a *Cirobu* gene, without 2019 ortholog in the second species, to one or more orthologs in this species.

Analysis of the quality of the results of our previous orthology assignment pipeline (24) indicated that orthologs of genes whose conserved domain extended over less than 40% of the protein sequence were frequently missed, as a result of the default threshold of the SiLiX software (30) used to build clusters of homologous proteins. This limitation was particularly problematic for a major class of developmental regulators, the transcription factors, whose conserved region is often limited to a short DNA-binding domain. To circumvent this issue, we adopted an iterative clustering procedure, starting with high-stringency SiLiX clustering and using progressively lower clustering stringency. Briefly, all genes from our 13 ascidian species, two echinoderms (*Acanthaster planci*, *Strongylocentrotus purpuratus*), two cephalochordates (*Branchiostoma lanceolatum, Branchiostoma belcheri*) and six vertebrates (*Homo sapiens*, *Mus musculus*, *Gallus gallus*, *Pelodiscus sinensis, Latimeria chalumnae*, *Callorhinchus milii*) were clustered at high stringency. Genes assigned to a family composed of genes from at least one echinoderm, six ascidians and four vertebrates were set aside. All other genes were again clustered, at a reduced stringency, and those assigned to a family with at least the same composition as above were set aside and the remaining genes were clustered at even more reduced stringency. This sequential procedure progressively built families from increasingly divergent genes. Ten stringency steps were used by tuning two SiliX parameters used to filter blast hits: -ident and -overlap (respectively, minimum of % identity, and minimum of % overlap between proteins, see supplementary methods). This new approach successfully increased the number of detected orthologs in each orthology class between ascidian species and with vertebrates, as illustrated on Figure 2 for *Ciona robusta*. Detection of one-to-many and many-to-many relationships was particularly improved. Detection of Human orthologs of *C. robusta* transcription factors was also strongly improved, as was the detection of TF orthologs in *Phallusia*, *Halocynthia* and *Molgula* (Supplementary Figure S1). Comparison to a manually-curated set of orthology relationships between *C. robusta* and *Homo sapiens* transcription factors (see supplementary methods) revealed a very high selectivity of the 2019 ANISEED orthology pipeline (88% of orthology relationships detected by the 2019 pipeline match the ground truth) as well as a 33% improvement in the number of detected orthology relationships between the 2017 and 2019 pipelines (Supplementary Figure S2).

As in the previous release, interactive phylogenetic trees are presented for each cluster. In addition, a specific tab in each gene card now lists for each gene its different classes of orthologs (one-to-one, one-to-many and many-to-many) in each of the 23 deuterostome reference species from which the clustering was built, with direct links to the gene card of the relevant database. The system's Genomicus synteny browser was also updated with these new relationships.

As in the previous release, functional gene annotation included conserved InterPro domains, the three most-related human genes, and Gene Ontology annotations. The latter were inherited from GO annotations of IPR domains, best human blast hits and orthologs as previously (24). In addition, this release now also provides annotations from a dedicated tunicate-specific GO Slim developed in the previous version, which are also mined by the ‘Genes (by GO term)’ search tool.

### Extension of the genomics and gene expression datasets

ANISEED 2017 Genomics datasets included staged RNA-seq for *C. robusta*, *P. mammillata* and *Halocynthia roretzi*, ChIP-seq for the H3K4me3 promoter mark in *C. robusta* and *P. mammillata* and SELEX-seq-based *in silico* transcription factor binding site prediction for *C. robusta* and *P. mammillata*. The major improvement in this release was the inclusion of a novel type of information, genome-wide chromatin accessibility status using ATAC-seq (31). In addition, we refined the TF binding site predictions and extended them to *Halocynthia*. Finally, we extended RNA-seq datasets to whole body regeneration experiments in *Botrylloides leachii*.

#### A dynamic view of embryonic chromatin accessibility by ATAC-Seq

Two datasets are available as public hubs in the Aniseed WashU browsers (32) for *C. robusta* and *P. mammillata*. In each species, the hub presents the normalized coverage values for ATAC-seq experiments carried out in WT embryos at the blastula (64-cell), early gastrula (112-cell), late gastrula and mid neurula stages as described in (33). Additional tracks present for WT *P. mammillata* embryos (16-cell, 32-cell) and for experimentally-perturbed 64- and 112-cell embryos from both species, in which the Wnt-ß-catenin pathway was activated by inhibition of the GSK3 kinase.

#### *In silico* prediction of conserved functional transcription factor binding sites

**Figure 3.**
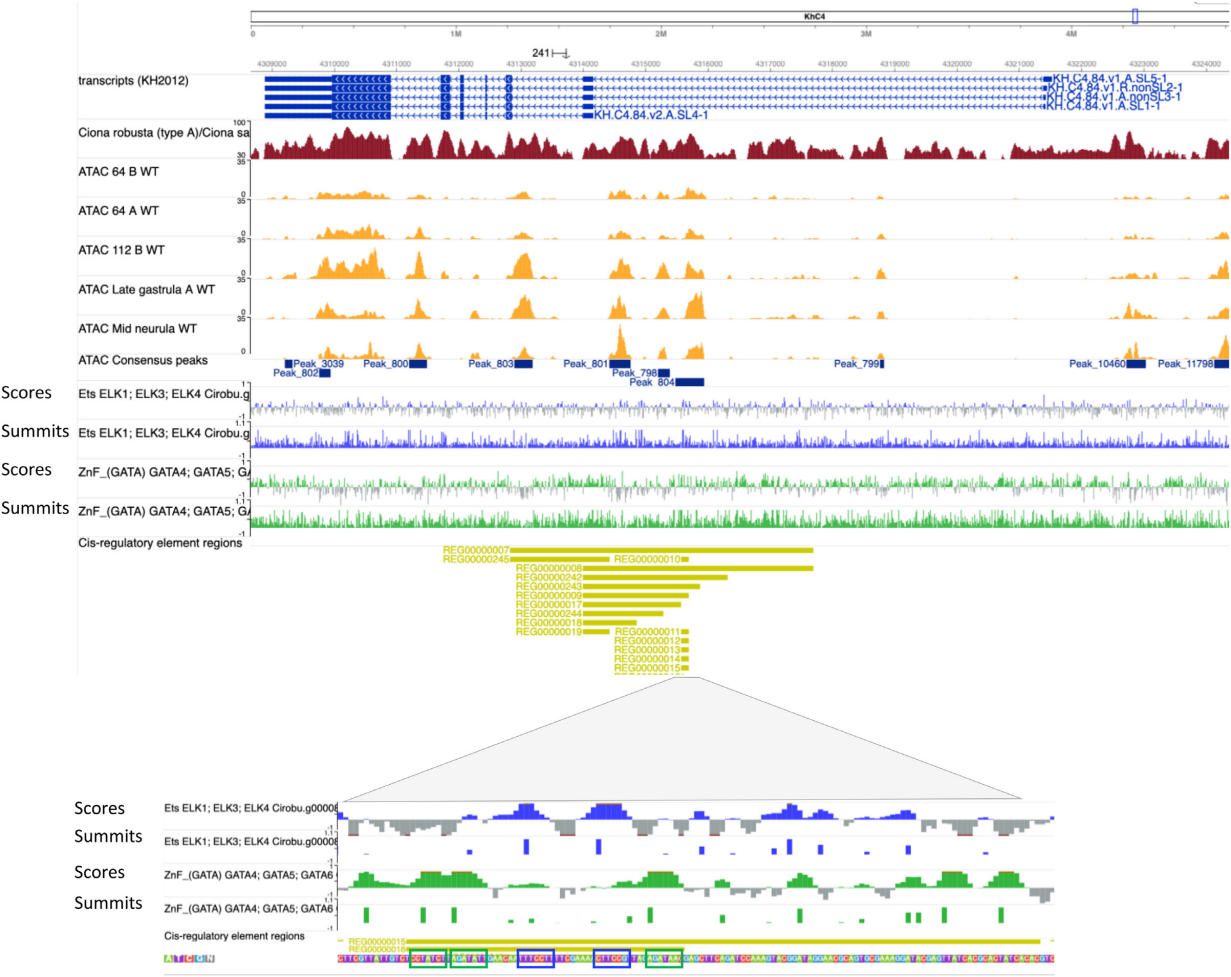
Visualization of *in silico* predicted transcription Factor binding sites based on SELEX-seq data at the *Otx* (*KH.C4.84*) locus in *Ciona robusta*. The top panel provides a global view of the organization of the locus, including exon positions, chormatin accessible regions, and TF-binding predictions. The bottom panel is an enlarged view of the *Otx* a-element (REG00000010), a short enhancer activated by ETS and GATA4/5/6 factors (35) through two ETS sites (blue boxes) and three GATA sites (green boxes). ‘Score’ indicated a continuous scoring of predicted affinity. ‘Summits’ associate the highest score of each peak to its summit base.

In the previous release, local scores corresponding to SELEX-eq based *in silico* predictions of the binding of 129 *C. robusta* transcription factors (34) and 84 *P. mammillata* orthologs were presented as public hubs of the WashU browsers of these species. We updated this dataset with the improved orthology detection pipeline, which increased the number of *Phallusia* orthologs to 107, and extended it to the 88 *H. roretzi* orthologs of the *Ciona* TFs. This dataset allows the visual identification of candidate binding sites, but the continuous nature of the score does not allow to programmatically identify putative binding sites. We therefore completed this dataset by extracting the summits corresponding to the center of peaks, associating to each of these summits the top score of the peak, and only keeping the top 10% of these summits to enrich for functional medium- to high-affinity binding sites. These candidate binding sites were highly predictive of functional binding sites. We extracted from the *C. robusta cis*-regulatory analysis section of ANISEED 320 experimentally identified TF binding sites, for which the structural TF class of the binding factor was known. 274 of these binding sites (85%) matched one the top 10% SELEX peaks of the expected structural class. Figure 3 illustrates on a well-characterized enhancer, the *Otx* a-element (35), that the combination of this dataset with chromatin accessibility maps is a powerful tool to identify the *cis*-regulatory logic driving development.

#### A transcriptomic analysis of whole-body regeneration

In addition to the transcriptional dynamics of embryonic development in solitary *C*., *Phallusia* and *Halocynthia* species, ANISEED 2019 now also includes a WashU public RNA-seq track hub showing the dynamics of *B. leachii* gene expression across 5 stages of whole-body regeneration, from a minuscule piece of vascular tissue, to a fully-grown adult colony.

#### Manually-curated expression data

ANISEED combines large-scale genomics information to smaller-scale experiments extracted from the literature and manually curated. Manual curation continued over the past 2 years, the main improvements consisting in a marked extension of the *P. mammillata* expression section (Supplementary Figure S3) and the manual curation of *C. robusta* expression datasets by *in situ* hybridization initially entered programmatically, leading to the removal of over four thousand expression profiles annotated ‘no expression’ or ‘whole embryo’ and conflicting either with the supporting evidence picture, or with higher-confidence datasets.

### New functionalities

#### Gene set extraction

The ‘Gene set’ view in the WashU genome browser offers the possibility to display several non-contiguous gene loci, including predefined lengths of 5′ and 3′ flanking sequences, in the same window. To further support this functionality, queries in ANISEED now support the extraction of lists of gene IDs that can be pasted into the ‘Add a new Gene set’ field of the WashU ‘Gene & region set’ App. To illustrate the process, Supplementary Figure S4 shows the expression by RNA-seq of 60 *B. leachii* genes annotated as Notch binding (**GO**:0005112). This overview identifies at a glance six genes with dynamic expression during Whole-body Regeneration, highlighting the potential of the approach to rapidly select members of a gene family with interesting expression, epigenetic profile, or presence of expected TF binding sites, depending on the scientific question addressed.

#### Online 4D visualization of gene expression profiles through MORPHONET

**Figure 4.**
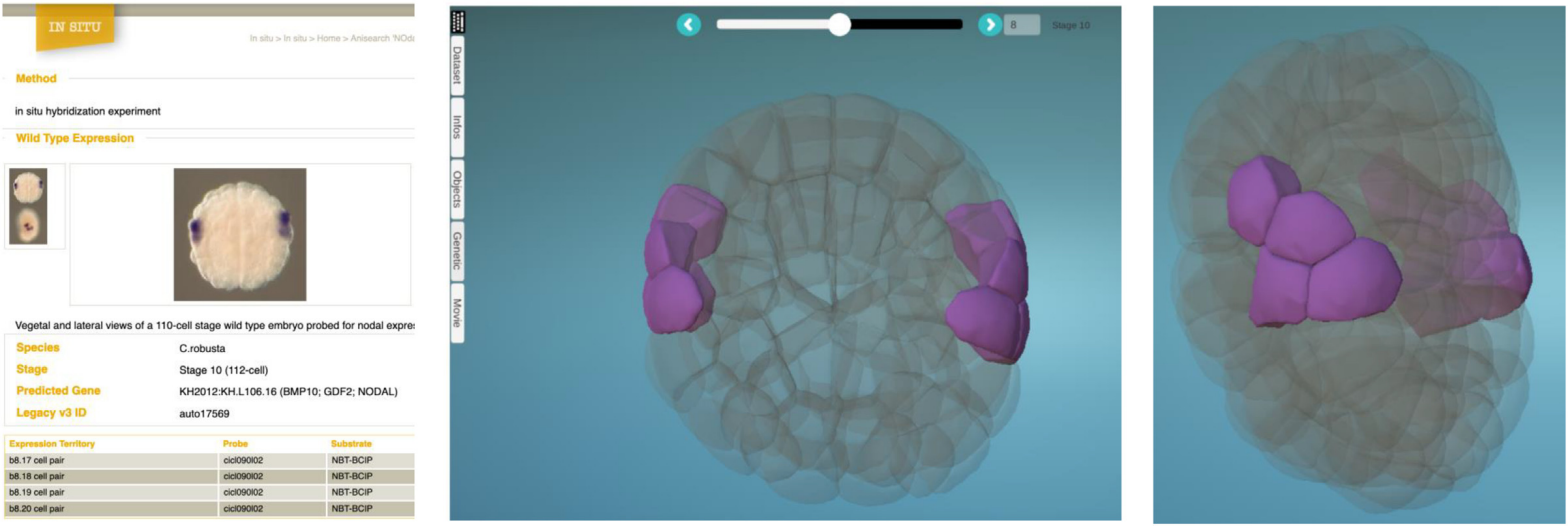
4D exploration of *in situ* hybridization expression patterns using the Morphonet browser. Left: Developmental browser display of the expression pattern of the *Ciona robusta Nodal* gene (KH.L106.16) at the early gastrula stage (Stage 10). Center and right: two views of the same experiment in the Morphonet browser.

ANISEED 2019 stores over 20 000 expression profiles by In situ hybridization. For some regulatory genes, >150 expression patterns have been collected from the literature, sometimes with discrepancies between experiments and authors. To facilitate the exploration of this dataset, we interfaced ANISEED to the Morphonet online morphogenetic browser (36). Each gene card includes a specific tab, which opens a Morphonet session to visualize the gene's expression pattern in 4D (Figure 4). Importantly, the Morphonet visualization summarizes all available WT expression patterns at a given stage, the density of the label of a given cell increasing with the proportion of experiments showing expression in this cell.

### Compatibility with FAIR guidelines and principles

Over the years, we have given particular attention to offer findable, accessible, interoperable and reusable data, in agreement with the FAIR guidelines. To fulfill this aim, ANISEED uses established international standards, when available, at all levels of its conception (Chado database schema; Gene Ontology, Sequence Ontology, ChEBI ontology; MISFISHIE, MINSEQE minimal information standards; InterPro database of protein families). In addition, necessary tunicate-specific ontologies and guidelines, such as the anatomical ontologies the Tunicate GO slim or the guidelines for nomenclature of genetic elements, are developed by the ANISEED biocurator team in collaboration with the relevant communities (37,38) and when applicable formatted according to the OBO Flat File Format.

All public data can be freely accessed and mined through web interfaces. In addition, an API (https://www.aniseed.cnrs.fr/api) and an extensive download section of files with standardized formats (https://www.aniseed.cnrs.fr/aniseed/download/download_data) are provided. ANISEED uses and provides standard formats for sharing data (JSON, Fasta, GFF3, GAF and NHX phylogenetic trees for example). Finally, all genomic elements in the database are retrievable by their unique identifier.

Tools are distributed under the GNU General Public License v3 (https://www.aniseed.cnrs.fr/aniseed/default/license). We are happy to share the code, currently deposited in a local Git server, with all interested scientists and to provide support for its installation. While ANISEED was initially developed for ascidians, a class of animals with stereotyped development, it can be used with minimal adaptation to any other taxon, for which an OBO anatomical ontology is available.

## Supplementary Material

gkz955_Supplemental_Files

## Notes

Present address: Paul Simion, Laboratory of Evolutionary Genetics and Ecology, URBE, NAXYS, University of Namur, Namur, Belgium.

Present address: Kazuhiro R. Nitta, Juntendo University, Graduate School of Medicine, Diagnostics and Therapeutics of Intractable Diseases Intractable Disease Research Center, Tokyo, Japan.

## References

[1] Delsuc F., Brinkmann H., Chourrout D., Philippe H. Tunicates and not cephalochordates are the closest living relatives of vertebrates. Nature. 2006; 439:965–968. 16495997 10.1038/nature04336

[2] Delsuc F., Tsagkogeorga G., Lartillot N., Philippe H. Additional molecular support for the new chordate phylogeny. Genes. 2008; 46:592–604. 10.1002/dvg.20450 19003928

[3] Kocot K.M., Tassia M.G., Halanych K.M., Swalla B.J. Phylogenomics offers resolution of major tunicate relationships. Mol. Phylogenet. Evol. 2018; 121:166–173. 29330139 10.1016/j.ympev.2018.01.005

[4] Delsuc F., Philippe H., Tsagkogeorga G., Simion P., Tilak M.-K., Turon X., López-Legentil S., Piette J., Lemaire P., Douzery E.J.P. A phylogenomic framework and timescale for comparative studies of tunicates. BMC Biol. 2018; 16:39. 29653534 10.1186/s12915-018-0499-2 PMC5899321

[5] Stolfi A., Ryan K., Meinertzhagen I.A., Christiaen L. Migratory neuronal progenitors arise from the neural plate borders in tunicates. Nature. 2015; 527:371–374. 26524532 10.1038/nature15758 PMC4654654

[6] Kaplan N., Razy-Krajka F., Christiaen L. Regulation and evolution of cardiopharyngeal cell identity and behavior: insights from simple chordates. Curr. Opin. Genet. Dev. 2015; 32:119–128. 25819888 10.1016/j.gde.2015.02.008 PMC4470797

[7] Stolfi A., Gainous T.B., Young J.J., Mori A., Levine M., Christiaen L. Early chordate origins of the vertebrate second heart field. Science. 2010; 329:565–568. 20671188 10.1126/science.1190181 PMC4970750

[8] Corbo J.C., Levine M., Zeller R.W. Characterization of a notochord-specific enhancer from the Brachyury promoter region of the ascidian, Ciona intestinalis. Development. 1997; 124:589–602. 9043074 10.1242/dev.124.3.589

[9] Imai K.S., Levine M., Satoh N., Satou Y. Regulatory blueprint for a chordate embryo. Science. 2006; 312:1183–1187. 16728634 10.1126/science.1123404

[10] Cao C., Lemaire L.A., Wang W., Yoon P.H., Choi Y.A., Parsons L.R., Matese J.C., Wang W., Levine M., Chen K. Comprehensive single-cell transcriptome lineages of a proto-vertebrate. Nature. 2019; 571:349–354. 31292549 10.1038/s41586-019-1385-y PMC6978789

[11] Stolfi A., Lowe E.K., Racioppi C., Ristoratore F., Brown C.T., Swalla B.J., Christiaen L. Divergent mechanisms regulate conserved cardiopharyngeal development and gene expression in distantly related ascidians. eLife. 2014; 3:e03728. 25209999 10.7554/eLife.03728 PMC4356046

[12] Hudson C., Ba M., Rouvière C., Yasuo H. Divergent mechanisms specify chordate motoneurons: evidence from ascidians. Dev. Camb. Engl. 2011; 138:1643–1652. 10.1242/dev.055426 21427146

[13] Razy-Krajka F., Stolfi A. Regulation and evolution of muscle development in tunicates. EvoDevo. 2019; 10:13. 31249657 10.1186/s13227-019-0125-6 PMC6589888

[14] Lowe E.K., Stolfi A. Developmental system drift in motor ganglion patterning between distantly related tunicates. Evodevo. 2018; 9:18. 30062003 10.1186/s13227-018-0107-0 PMC6057086

[15] Kassmer S.H., Rodriguez D., De Tomaso A.W. Colonial ascidians as model organisms for the study of germ cells, fertility, whole body regeneration, vascular biology and aging. Curr. Opin. Genet. Dev. 2016; 39:101–106. 27379900 10.1016/j.gde.2016.06.001

[16] Tiozzo S., Brown F., Tomaso A.W. Regeneration and stem cells in ascidians. Stem Cells. 2008; Netherlands Springer 95–112.

[17] Zondag L.E., Rutherford K., Gemmell N.J., Wilson M.J. Uncovering the pathways underlying whole body regeneration in a chordate model, Botrylloides leachi using de novo transcriptome analysis. BMC Genomics. 2016; 17:114. 26879048 10.1186/s12864-016-2435-6 PMC4755014

[18] Gorsky G., Youngbluth M.J., Deibel D. Response of Marine Ecosystems to Global Change: Ecological Impact of Appendicularians. Éditions Scientifiques. 2005; Paris 435.

[19] Colarusso P., Nelson E., Ayvazian S., Carman M., Chintala M., Grabbert S., Grunden D. Quantifying the ecological impact of invasive tunicates to shallow coastal water systems. Manag. Biol. Invasions. 2016; 7:33–42.

[20] Sato A., Kawashima T., Fujie M., Hughes S., Satoh N., Shimeld S.M. Molecular basis of canalization in an ascidian species complex adapted to different thermal conditions. Sci. Rep. 2015; 5:16717. 26577490 10.1038/srep16717 PMC4649386

[21] Vered G., Kaplan A., Avisar D., Shenkar N. Using solitary ascidians to assess microplastic and phthalate plasticizers pollution among marine biota: a case study of the Eastern Mediterranean and Red Sea. Mar. Pollut. Bull. 2019; 138:618–625. 30660313 10.1016/j.marpolbul.2018.12.013

[22] Tassy O., Dauga D., Daian F., Sobral D., Robin F., Khoueiry P., Salgado D., Fox V., Caillol D., Schiappa R. et al. The ANISEED database: digital representation, formalization, and elucidation of a chordate developmental program. Genome Res. 2010; 20:1459–1468. 20647237 10.1101/gr.108175.110 PMC2945195

[23] Brozovic M., Martin C., Dantec C., Dauga D., Mendez M., Simion P., Percher M., Laporte B., Scornavacca C., Di Gregorio A. et al. ANISEED 2015: a digital framework for the comparative developmental biology of ascidians. Nucleic Acids Res. 2016; 44:D808–D818. 26420834 10.1093/nar/gkv966 PMC4702943

[24] Brozovic M., Dantec C., Dardaillon J., Dauga D., Faure E., Gineste M., Louis A., Naville M., Nitta K.R., Piette J. et al. ANISEED 2017: extending the integrated ascidian database to the exploration and evolutionary comparison of genome-scale datasets. Nucleic Acids Res. 2018; 46:D718–D725. 29149270 10.1093/nar/gkx1108 PMC5753386

[25] Nguyen N.T.T., Vincens P., Roest Crollius H., Louis A. Genomicus 2018: karyotype evolutionary trees and on-the-fly synteny computing. Nucleic Acids Res. 2018; 46:D816–D822. 29087490 10.1093/nar/gkx1003 PMC5753199

[26] Jeffery W.R., Swalla B.J. An evolutionary change in the muscle lineage of an anural ascidian embryo is restored by interspecific hybridization with a urodele ascidian. Dev. Biol. 1991; 145:328–337. 2040375 10.1016/0012-1606(91)90131-l

[27] Blanchoud S., Rutherford K., Zondag L., Gemmell N.J., Wilson M.J. De novo draft assembly of the Botrylloides leachii genome provides further insight into tunicate evolution. Sci. Rep. 2018; 8:1–18. 29615780 10.1038/s41598-018-23749-w PMC5882950

[28] Blanchoud S., Rinkevich B., Wilson M.J. Kloc M, Kubiak JZ Whole-Body regeneration in the colonial tunicate botrylloides leachii. Marine Organisms as Model Systems in Biology and Medicine, Results and Problems in Cell Differentiation. 2018; Cham Springer International Publishing 337–355. 10.1007/978-3-319-92486-1_16 30083927

[29] Nishida H. Development of the appendicularian Oikopleura dioica: culture, genome, and cell lineages. Dev. Growth Differ. 2008; 50(Suppl. 1):S239–S256. 18494706 10.1111/j.1440-169X.2008.01035.x

[30] Miele V., Penel S., Duret L. Ultra-fast sequence clustering from similarity networks with SiLiX. BMC Bioinformatics. 2011; 12:116. 21513511 10.1186/1471-2105-12-116 PMC3095554

[31] Buenrostro J.D., Giresi P.G., Zaba L.C., Chang H.Y., Greenleaf W.J. Transposition of native chromatin for fast and sensitive epigenomic profiling of open chromatin, DNA-binding proteins and nucleosome position. Nat. Methods. 2013; 10:1213–1218. 24097267 10.1038/nmeth.2688 PMC3959825

[32] Zhou X., Lowdon R.F., Li D., Lawson H.A., Madden P.A.F., Costello J.F., Wang T. Exploring long-range genome interactions using the WashU Epigenome Browser. Nat. Methods. 2013; 10:375–376. 23629413 10.1038/nmeth.2440 PMC3820286

[33] Madgwick A., Magri M.S., Dantec C., Gailly D., Fiuza U.-M., Guignard L., Hettinger S., Gomez-Skarmeta J.L., Lemaire P. Evolution of embryonic cis-regulatory landscapes between divergent Phallusia and Ciona ascidians. Dev. Biol. 2019; 448:71–87. 30661644 10.1016/j.ydbio.2019.01.003

[34] Nitta K.R., Vincentelli R., Jacox E., Cimino A., Ohtsuka Y., Sobral D., Satou Y., Cambillau C., Lemaire P. Vincentelli R High-Throughput protein production combined with High- Throughput SELEX identifies an extensive Atlas of ciona robusta transcription factor DNA-binding specificities. High-Throughput Protein Production and Purification: Methods and Protocols, Methods in Molecular Biology. 2019; NY Springer 487–517. 10.1007/978-1-4939-9624-7_23 31267468

[35] Bertrand V., Hudson C., Caillol D., Popovici C., Lemaire P. Neural tissue in ascidian embryos is induced by FGF9/16/20, acting via a combination of maternal GATA and Ets transcription factors. Cell. 2003; 115:615–627. 14651852 10.1016/s0092-8674(03)00928-0

[36] Leggio B., Laussu J., Carlier A., Godin C., Lemaire P., Faure E. MorphoNet: an interactive online morphological browser to explore complex multi-scale data. Nat. Commun. 2019; 10:1–8. 31249294 10.1038/s41467-019-10668-1 PMC6597584

[37] Manni L., Gasparini F., Hotta K., Ishizuka K.J., Ricci L., Tiozzo S., Voskoboynik A., Dauga D. Ontology for the asexual development and anatomy of the colonial chordate botryllus schlosseri. PLoS One. 2014; 9:e96434. 24789338 10.1371/journal.pone.0096434 PMC4006837

[38] Stolfi A., Sasakura Y., Chalopin D., Satou Y., Christiaen L., Dantec C., Endo T., Naville M., Nishida H., Swalla B.J. et al. Guidelines for the nomenclature of genetic elements in tunicate genomes. Genesis. 2015; 53:1–14. 25220678 10.1002/dvg.22822 PMC4308547

