# Supplementary material for: ANISEED 2019: 4D exploration of genetic data for an extended range of tunicates
Source: Nucleic Acids Res. 2019 Nov 4;48(D1):D668–75. doi: 10.1093/nar/gkz955 (PMC7145539; doi:10.1093/nar/gkz955)
Supplement: gkz955_Supplemental_Files [file gkz955_supplemental_files.zip › Supplementary Online material-Dardaillon-2019.pdf]

ANISEED 2019: 4D exploration of genetic data for an extended range of tunicates

Justine Dardaillon et al.

SUPPLEMENTARY ONLINE MATERIAL

Supplementary methods 1
Supplementary References 2
Authors contributions 3
Supplementary figures 4
Annexes 7

Supplementary methods

Automated orthology assignment

The ten iterative stringency steps corresponding to the tuning of two SiliX parameters used to filter blast hits: --ident and --overlap (respectively, minimum of % identity, and minimum of % overlap between proteins, see supplementary data) as follows:

Step 1: r1_i=0.60; r1_r=0.40 (≥ 60% protein identity, ≥40% of the length of the ORFs aligned)
Step 2: r2_i=0.50; r2_r=0.35
Step 3: r3_i=0.44; r3_r=0.25
Step 4: r4_i=0.38; r4_r=0.20
Step 5: r5_i=0.35; r5_r=0.15
Step 6: r6_i=0.32; r6_r=0.15
Step 7: r7_i=0.29; r7_r=0.10
Step 8: r8_i=0.26; r8_r=0.10
Step 9: r9_i=0.23; r9_r=0.10
Step 10: r10_i=0.20; r10_r=0.10).

Manual assignments of Human orthologs to *Ciona robusta* transcription factors

Previous reports identified 669 transcription factors and transcription-associated factors in *Ciona robusta* (1, 2). This list was extended based on the functional annotation in ANISEED of the KH2008 *Ciona*

robusta gene model set (3). The annotated KH2008 genes were scanned for specific Gene Ontology (GO) terms and InterPro domains associated to transcriptional regulation (see Annex 2). We excluded RNA polymerase I/III TFs and selected DNA-binding TF with highest protein sequence similarity to well-characterized vertebrate transcription factors, producing a list of 338 core *Ciona* transcription factors (Annex1). A list of 1591 core or possible Human transcription factors was extracted from Suppl S3 from (4), from which we only selected the factors classified as 'a', 'b', 'c' or 'other'.

We selected the longest protein for each *Ciona robusta* (formerly *Ciona intestinalis* type A*)* core transcription factor and putative human transcription factors and all proteins were run through the batch mode of CDD search and scripts were designed to extract the indicated DNA-binding domain(s) from each factor (see Annex 2).

Previous phylogeny work was reported on bHLH factors (5); Fox, ETS, nuclear receptors and NFkappaB (6); HMG, bZIP and Zinc finger genes (7); and Wnt, Hh Jak/Stat (8). These orthology relationships were confirmed and extended in light of (4). Tree-based phylogenies between *Ciona* and human genes were built for core transcription factors of the Arid, AP2, bHLH, bZIP, ETS, Forkhead, HMG, Homeodomain, IPT/TIG, IRF, MADS, Paired box, RFX, T-box, SP/klf C2H2 zinc fingers and Gli/Zic C2H2 zinc finger families. Phylogenetic trees of the DNA-binding domains were built using Seaview (alignment with Muscle, tree building with PhyML, using default parameters and the LG model) (9). ENSEMBL (releases 65 and 68) orthology relationships were inherited via Biomart for other core transcription factors. Altogether this process identified 291 core transcription factors with human orthologs, establishing 734 orthology relationships to human genes (see Annex 3).

Supplementary References

1. Imai,K.S., Hino,K., Yagi,K., Satoh,N. and Satou,Y. (2004) Gene expression profiles of transcription factors and signaling molecules in the ascidian embryo: towards a comprehensive understanding of gene networks. *Development*, **131**, 4047–58.

2. Miwata,K., Chiba,T., Horii,R., Yamada,L., Kubo,A., Miyamura,D., Satoh,N. and Satou,Y. (2006) Systematic analysis of embryonic expression profiles of zinc finger genes in Ciona intestinalis. *Dev. Biol.*, **292**, 546–554.

3. Satou,Y., Mineta,K., Ogasawara,M., Sasakura,Y., Shoguchi,E., Ueno,K., Yamada,L., Matsumoto,J., Wasserscheid,J., Dewar,K., *et al.* (2008) Improved genome assembly and evidence-based global gene model set for the chordate Ciona intestinalis: new insight into intron and operon populations. *Genome Biol.*, **9**, R152.

4. Vaquerizas,J.M., Kummerfeld,S.K., Teichmann,S.A. and Luscombe,N.M. (2009) A census of human transcription factors: function, expression and evolution. *Nat. Rev. Genet.*, **10**, 252–263.

5. Satou,Y., Imai,K.S., Levine,M., Kohara,Y., Rokhsar,D. and Satoh,N. (2003) A genomewide survey of developmentally relevant genes in Ciona intestinalis. I. Genes for bHLH transcription factors. *Dev. Genes Evol.*, **213**, 213–221.

6. Yagi,K., Satou,Y., Mazet,F., Shimeld,S.M., Degnan,B., Rokhsar,D., Levine,M., Kohara,Y. and Satoh,N. (2003) A genomewide survey of developmentally relevant genes in Ciona intestinalis. III. Genes for Fox, ETS, nuclear receptors and NFkappaB. *Dev. Genes Evol.*, **213**, 235–244.

7. Yamada,L., Kobayashi,K., Degnan,B., Satoh,N. and Satou,Y. (2003) A genomewide survey of developmentally relevant genes in Ciona intestinalis. IV. Genes for HMG transcriptional regulators, bZip and GATA/Gli/Zic/Snail. *Dev. Genes Evol.*, **213**, 245–253.

8. Hino,K., Satou,Y., Yagi,K. and Satoh,N. (2003) A genomewide survey of developmentally relevant genes in Ciona intestinalis. VI. Genes for Wnt, TGFbeta, Hedgehog and JAK/STAT signaling pathways. *Dev. Genes Evol.*, **213**, 264–272.

9. Gouy,M., Guindon,S. and Gascuel,O. (2010) SeaView version 4: A multiplatform graphical user interface for sequence alignment and phylogenetic tree building. *Mol. Biol. Evol.*, **27**, 221–224.

Authors contributions

JD developed and updated the ANISEED database and interfaces.

DD, the ANISEED biocurator, integrated expression data produced by LB, WR, SF, RD and MV, with help from MF and MG-B.

PS and ED developed the refined orthology pipeline, which was tested against a manually-curated set of orthology relationships between *Ciona robusta* and *Homo sapiens* TFs, produced by KRN.

EF integrated ANISEED with Morphonet, with help from JD.

TO, KW and HN sequenced and assembled the *Oikopleura dioica* genome and generated a gene model set.

MBDB, JFR and BD sequenced and assembled the *Corella inflata* genome and generated a gene model set.

AL and HRC updated the Genomicus server for tunicates with information provided by PS and CD.

MN and J-NV updated the transposable element annotations.

CD set up and updated the WashU genome browsers of the system, curated gene model datasets and functionally annotated the *Oikopleura* and *Corella* gene models provided by HN and BD.

CD and PL headed the project, secured its funding and prepared the manuscript.

Supplementary figures

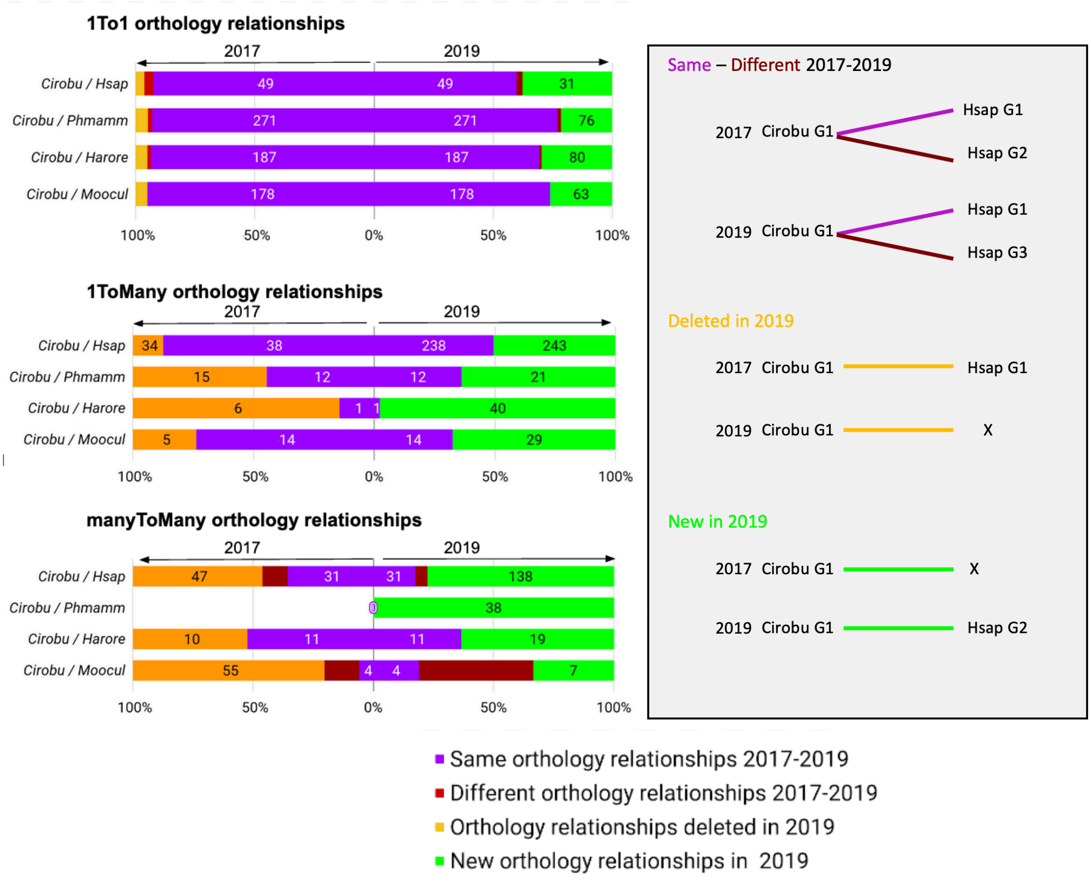

Supplementary Figure 1: Comparison of the 1-to-1, 1-to-many and many-to-many orthology relationships detected in the ANISEED 2017 and 2019 releases for a list of 579 *Ciona robusta* transcription factors annotated as "RNA polymerase II regulatory region sequence-specific DNA binding" (**GO**:0000977) (See Annex 1). Each half of the bar graphs present the analysis in 2017 (left half) and 2019 (right half) of the percentage (and number) of orthology relationships linking one *Ciona robusta (Cirobu*) gene to one gene in the second species indicated (*Hasp: Homo sapiens ; Phmamm: Phallusia mammillata; Harore: Halocynthia roretzi; Moocul; Molgula oculata)*. Four scenarios are distinguished, as illustrated on the right side of the figure. Same orthology relationships 2017-2019: orthology relationships found in the 2017 and 2019 orthology pipelines. Different orthology relationships 2017-2019: the *Cirobu* gene has orthologs in the second species according to both pipelines, but these differ. New orthology relationships in 2019: 2019 orthology relationships linking a *Cirobu* gene, without 2017 ortholog in the second species, to one or more orthologs in this species. Deleted orthology relationships in 2019: 2017 orthology relationships linking a *Cirobu* gene, without 2019 ortholog in the second species, to one or more orthologs in this species.

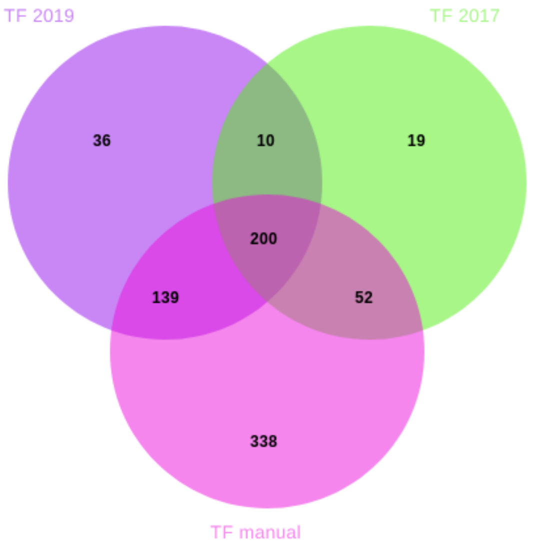

Supplementary Figure 2: Comparison of the orthology relationships found between a core set of 291 *Ciona robusta* transcription factors and their human counterparts (Annexes 2, 3). TF 2017 (green): orthology relationships predicted by the 2017 Aniseed pipeline; TF 2019 (mauve): orthology relationships predicted by the 2019 pipeline; TF manual (Pink): manually curated orthology relationships (see supplementary methods). Note the high selectivity of the pipeline (88% of the 2019 orthology relationships match the manually curated set) and the higher proportion of manually-curated relationships detected by the 2019 pipeline (2019: 46%; 2017: 34%). Reasons that could explain that some manually-curated relationships are not found by the pipeline include: 1 - a higher stringency in calling orthologs by the pipeline, which flattens branches with less than 30% bootstrap support; 2 – The use of only two species in the manual curation sample, which may fail to identify some speciation and gene loss events; 3 – a tendency of the Aniseed pipeline to place distant ascidian paralogs into different clusters.

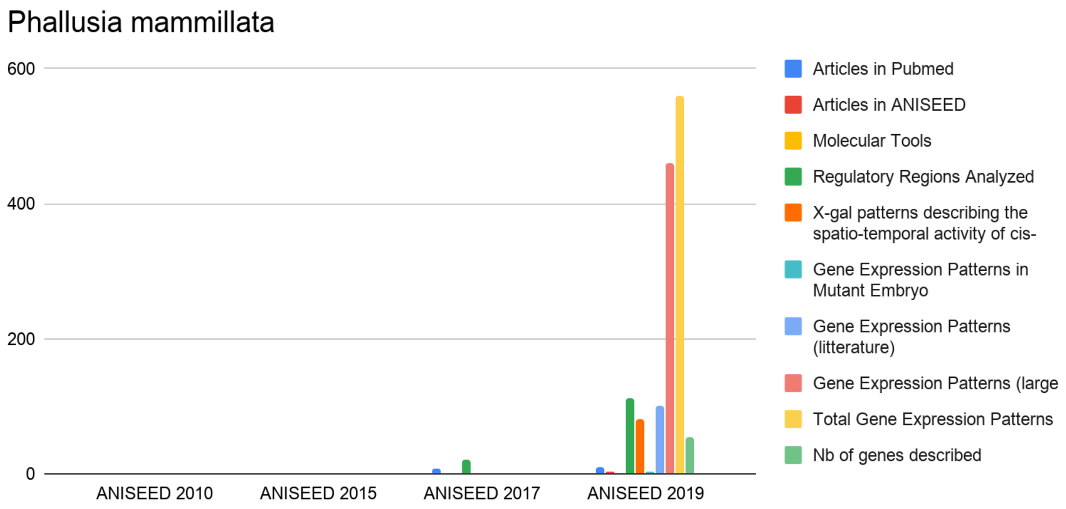

Supplementary Figure 3: Evolution in successive ANISEED releases of the volume of various types of *Phallusia mammillata* data. Note the marked increase in the 2019 release.

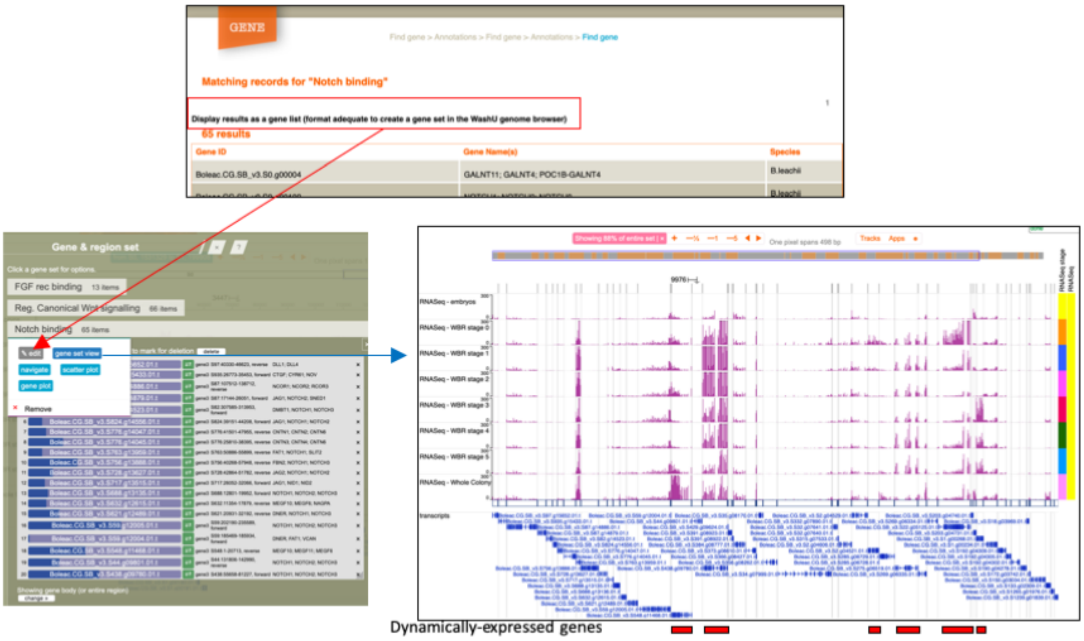

Supplementary Figure 4: Construction of a gene set in the developmental browser and visualization of its expression profiles using the "Gene set view" of the WashU browser. Top: results page of a search for *Ciona robusta* genes annotated with the GO term "Notch binding" (GO:0005112). Top: the boxed line allows to download the unique Gene IDs for all 66 genes annotated as "Notch binding". Upon pasting this gene list in the "Gene and region set" field of the WashU browser (bottom left), a new gene set is created whose expression can be visualized with the "Gene set view" (bottom right). Several gene sets can be created, here "Notch binding", "FGF receptor binding" and "Regulation of canonical Wnt signaling".

Annexes

Annex 1: list of 579 *Ciona robusta* genes annotated (Tunicate GOslim) with the term "RNA polymerase II regulatory region sequence-specific DNA binding" (**GO**:0000977) and used in Supplementary Figure 1A.

Annex 2: Manually curated list of core *Ciona robusta* transcription factors used in Supplementary Figure 1B, with DNA binding domains and GO terms.

Annex 3: Manually-curated orthology relationships established by core *Ciona robusta* transcription factors in *Homo sapiens*.
